# An Erring Witness

**Published:** 1876-10

**Authors:** 


### *AN ERRING WITNESS.*

Somebody connected with the Daily and Weekly *Witness* of this city, is undertaking to sit in final judgment upon the researches of scientific men upon food-topics, to such an extent as to exclude from the pages of these papers the advertisements of manufacturers of improved foods. A most remarkable case of bigotry of this nature has been related to us by our intelligent and earnest neighbors—the Health Food Company.

Mr. Elmendorf, the advertising agent of the *Witness*, called recently on the Health Food Company, and solicited an advertisement. Terms were agreed upon and an advertisement written, headed

AVOID "GRAHAM!"

The advertisement went on to state that the Company had proved that "Graham" flour, crushed wheat, grits, and the like were pernicious foods, and offered to send printed matter in support of the assertion to all who might apply.

The next day Mr. Elmendorf returned and announced that the *Witness* refused to publish the advertisement on the ground that to do so, would be to deny the editorial teachings of the paper, and to state a falsehood. A modification of the language was demanded by the *Witness*, and most emphatically refused by the Health Food Company.

It is difficult to see precisely upon what principle of decency the *Witness* people can exclude the advertisement of this Company from its pages. If any one thing has been shown beyond the possibility of refutation, by the efforts and researches of this organization, it is that the best products which pass under the name of "Graham" flour, are pestiferous, cruel, and devastating food for humanity. In the same category may be classed all the crushed and cracked cereals which have not been denuded of their hully bran by the wet process. To eat them habitually, is to so deplete the system as to make it a ready prey of dangerous disease; to arrest the peristaltic motion of the stomach; to render the emulsification of all food imperfect and ineffectual; to drain the whole intestinal canal of vital fluid; to compel the most dangerous form of hemorrhoids; to induce fatal anemic conditions, in short—to destroy human life.

Now, the old "Graham" humbug is completely exploded, and no man who claims to be a teacher, a manufacturer of public sentiment, an educator of mankind, has the smallest right to be ignorant of the fact. Especially has he no right to withhold light of such vast importance from others, however benighted or bigoted he may be himself.

We do not wish to dwell on this theme, because we have little patience with stupidity wherever it exists, but we will add that the day has well nigh passed when bran is to be employed as food. It is flinty, insoluble, indigestible; and while some thick-skinned persons—like the Indian and the negro—can survive its continued use, it is clearly harmful in the large majority of cases. Graham was wise enough to observe that the dark, soluble part of the grain was the best part of it, but he did not know that this good portion could be separated from the hully bran. So he directed that the whole thing be swallowed, and hence a multitude of poor creatures have struggled or died miserably, because of the teachings of this false prophet of hygiene. Let us hope that there are few public instructors who will be found guilty of advocating this ancient and exploded error.

